# Effect of Adding Human Umbilical Cord Mesenchymal Stem Cells–Derived Secretome on Sperm Quality Improvement by Swim‐Up Method

**DOI:** 10.1155/ogi/8181670

**Published:** 2026-02-24

**Authors:** Binarwan Halim, Cynthia Retna Sartika, Laura Agnes Edessa Sibuea, Carine Annabella Widjaja, Diana Novia, Rima Haifa, Karina Kalasuba

**Affiliations:** ^1^ Department of Obstetrics and Gynecology, Faculty of Medicine, Universitas Prima Indonesia, Medan City, 20118, North Sumatera, Indonesia, unprimdn.ac.id; ^2^ Department of Obstetrics and Gynecology, Faculty of Medicine, Universitas Sumatera Utara, Medan City, 20155, North Sumatera, Indonesia, usu.ac.id; ^3^ Halim Fertility Center, Stella Maris Women’s and Children’s Hospital, Medan City, North Sumatera, 20152, Indonesia; ^4^ Department of Pharmacology and Clinical Pharmacy, Faculty of Pharmacy, Universitas Padjadjaran, Sumedang Regency, 45363, West Java, Indonesia, unpad.ac.id; ^5^ Prodia StemCell Indonesia, Central Jakarta City, 10430, DKI Jakarta, Indonesia

**Keywords:** DNA fragmentation, secretome, sperm quality, swim up

## Abstract

**Background:**

Suboptimal sperm quality often poses a challenge to successful fertilization. This study hypothesizes that secretome could enhance sperm quality and increase the likelihood of successful pregnancy. This research aims to assess the impact of secretome on various sperm quality parameters, including concentration, motility, and DNA fragmentation, while considering patient‐related factors such as age, duration of abstinence, and body mass index (BMI).

**Method:**

An analysis involving 45 patients enrolled in the pregnancy program at Halim Fertility Center, Stella Maris Women’s and Children’s Hospital, Indonesia, from April to September 2023 was conducted. Semen samples from these patients were subjected to the swim‐up method and divided into two groups: Group A, which underwent swim‐up without secretome, and Group B, which underwent swim‐up with the addition of secretome. DNA fragmentation analysis was performed on the sperm swim‐up results from both groups. The sperm analysis data obtained before swim‐up (pretreatment) with those of Group A and Group B were compared.

**Result:**

The demographic data revealed an average age of 37.67 ± 5.36 years, abstinence duration of 4.00 ± 1.15 days, and BMI of 28.20 ± 3.49 kg/m^2^ among the patients. No significant difference was observed in sperm concentration between pretreatment, Group A, and Group B (mil/mL) (36.2 ± 18.5; 36.3 ± 18.4; 36.6 ± 19.3). However, a significant difference was found in the rapid progressive motility of sperm across pretreatment, Group A, and Group B (%) (0.48 ± 1.32; 13.7 ± 8.3; 17 ± 8.3), as well as a significant reduction in DNA fragmentation in Group B compared to Group A (%) (3.48 vs. 4.39).

**Conclusions:**

The findings suggest that secretome enhances rapid progressive motility and reduces DNA fragmentation rates without affecting sperm concentration.

## 1. Introduction

Infertility is a common condition impacting the reproductive system, characterized by a couple’s failure to conceive after 12 months or more of regular, unprotected sexual intercourse, or an individual’s inability to achieve reproduction [1]. This condition is multifactorial, potentially resulting from male or female factors, a combination of both, or remaining unexplained. Male factor infertility constitutes around 20% of infertility cases, with 30% resulting from a combination of male and female factors. Male factor infertility varies in severity, influenced by sperm parameters including motility, concentration, morphology, and the DNA fragmentation index (DFI), which may be affected by reactive oxygen species (ROS) [2]. Suboptimal sperm quality impedes fertilization and negatively influences embryonic development and outcomes in assisted reproductive technologies (ARTs), including in vitro fertilization (IVF) [3].

Currently, the treatment options for male factor infertility include intrauterine insemination (IUI), IVF, surgical procedures, and pharmacological interventions. These strategies are designed to improve sperm quality and mitigate underlying causes to improve pregnancy outcomes. Based on the available evidence, there is a positive correlation between treatment success rates and improved sperm quality, which emphasizes the necessity of effective sperm processing techniques [4]. Standard sperm processing methods, including swim‐up, density gradient centrifugation, and simple rinsing, are designed to enhance the quality and functionality of sperm during insemination and IVF procedures. However, these methods primarily rely on the isolation of intrinsically high‐quality sperm, without addressing the underlying injury or oxidative stress [5]. To overcome these constraints, recent developments in cell therapy, particularly the utilization of secretomes, have demonstrated potential for enhancing the quality of sperm. The secretome, a complex assemblage of bioactive molecules, including cytokines and growth factors, has been shown to facilitate cellular repair and regeneration in a variety of biological contexts. By reducing DNA fragmentation, improving morphology, enhancing motility, and mitigating ROS‐induced damage, secretome is garnering attention for its potential to rejuvenate sperm quality. In ART, the probability of effective fertilization and clinical pregnancy could be substantially elevated by these effects [3]. It is the objective of this investigation to assess the influence of secretome on critical sperm parameters, such as concentration, motility, morphology, and DFI. In addition, the investigation will evaluate the impact of patient‐related variables, including age, abstinence duration, and body mass index (BMI), to offer a thorough comprehension of the variables that influence sperm quality and ART outcomes.

## 2. Materials and Methods

A retrospective analysis was conducted on 46 male patients enrolled in the pregnancy program at Stella Maris Women’s and Children’s Hospital, Indonesia, Halim Fertility Center, from April to September 2023. The study involved couples undergoing infertility treatment, with male participants selected using a consecutive sampling method based on inclusion and exclusion criteria. Eligible participants were male aged ≤ 50 years, while those diagnosed with severe oligoasthenoteratozoospermia, cryptozoospermia, or azoospermia were excluded. Patients with infectious diseases such as hepatitis B (HBsAg), hepatitis C (HCV), or HIV were also excluded. Primary data were collected using a structured questionnaire containing information on the participant’s medical record number, name, address, age, contact number, weight, height, blood type, history of smoking and alcohol consumption, duration of sexual abstinence, and sample collection time.

Semen samples were collected in antiseptic containers after 3–5 days of sexual abstinence. After 30–60 min of liquefaction on a mixer, samples were pipetted to ensure homogeneity and volume was measured. A 10 μL aliquot of the semen was analyzed using a Makler Sperm Counting Chamber according to the *World Health Organization Laboratory Manual for the Examination and Processing of Human Semen (*6th Edition, 2021). Prior to sperm processing, DNA integrity was assessed using the Sperm Chromatin Dispersion (SCD) assay with the Lenshooke R10 Sperm DNA Fragmentation Test Kit. The DFI was evaluated under a 40x microscope, where sperm exhibiting fragmented DNA were identified by a distinct “halo zone,” reflecting the degree of DNA damage.

The mesenchymal stem cell (MSC)–derived secretome used in this study was obtained from the GMP‐certified laboratory of PT Prodia StemCell Indonesia (ProSTEM, Jakarta, Indonesia). The product was manufactured, characterized, and quality controlled under GMP standards, and its specifications are documented in a certificate of analysis (CoA) provided by ProSTEM. The CoA includes total protein concentration, concentrations of key growth factors and cytokines, and sterility, endotoxin, and batch‐to‐batch consistency data verified under GMP quality assurance. All batches used in this study met specification limits and passed sterility and endotoxin testing prior to use. For the experimental procedure, the remaining semen was mixed with 1 mL of G‐MOPS™ PLUS (Vitrolife) buffer and divided into two equal portions and then centrifuged at a relative centrifugal force (RCF) of 1400 × *g* for 10 min, with the supernatant discarded. Group A underwent the swim‐up procedure without secretome, while Group B underwent swim‐up with the addition of secretome, prepared by mixing one‐part secretome with nine parts of G‐MOPS™ PLUS buffer (1:9).

**Figure 1:**
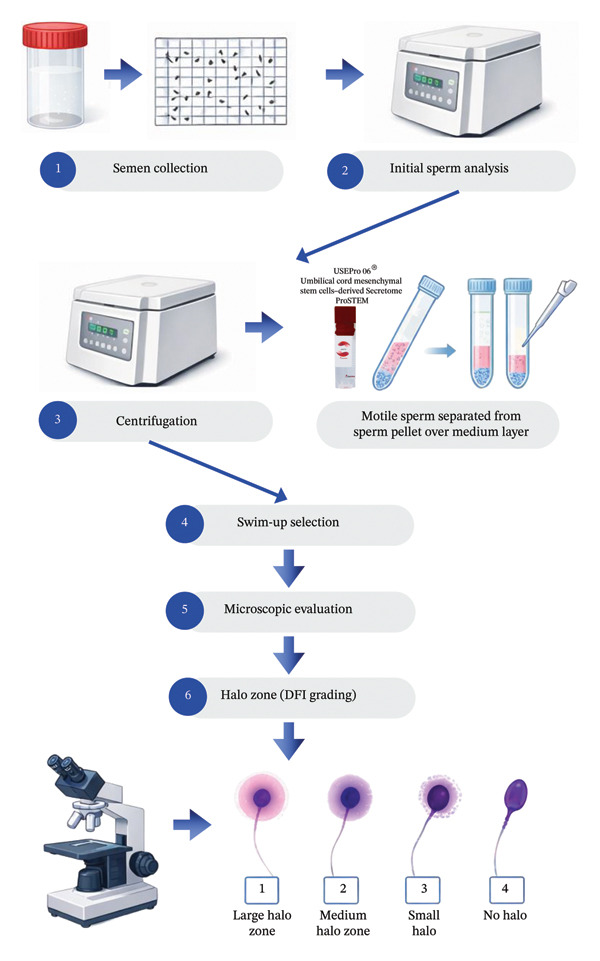
Schematic representation of the experimental procedure for evaluating the effect of secretome on sperm quality.

For the swim‐up technique, 1 mL of G‐MOPS™ PLUS buffer (Group A) and G‐MOPS™ PLUS buffer with secretome (Group B) were carefully layered over each pellet. The tubes were incubated at 37 °C for 45 min at a 45° angle. After incubation, the supernatant containing motile sperm was gently aspirated and subsequently centrifuged at 1400 × g for 5 min. The resulting pellet, containing the selected motile sperm population, was resuspended in 0.3 mL of buffer for subsequent sperm analysis. The same procedures were used for both groups before and after sperm processing, and DFI values were evaluated for comparison, as illustrated in Figure 1.

Data were analyzed using IBM SPSS Statistics Version 29.0.2.0 (IBM Corp., Armonk, NY, USA). Continuous variables were expressed as mean ± standard deviation (SD). Normality of data distribution was verified using the Shapiro–Wilk test, and homogeneity of variance was assessed with Levene’s test. One‐way analysis of variance (ANOVA) was used to compare sperm parameters (concentration, motility, morphology, and DFI) among pretreatment, Group A (swim‐up without secretome), and Group B (swim‐up with secretome). When significant differences were found, Duncan’s post hoc test was applied for pairwise comparisons. A *p* value < 0.05 was considered statistically significant.

## 3. Result

The demographic data indicated that the mean age of the participants was 37.67 ± 5.36 years, with an average abstinence duration of 4.00 ± 1.15 days and a mean BMI of 28.20 ± 3.49 kg/m^2^, as shown in Table 1. The comparison of sperm quality among pretreatment, Group A (control), and Group B (secretome addition) is summarized in Table 2. Sperm concentration did not differ significantly among the three groups (*p* = 0.995). However, significant improvements were observed in rapid progressive (RP) motility (Category A) (*p* < 0.001) and total motility (TM) (A + B) (*p* < 0.001) following secretome addition, while the proportion of nonprogressive and immotile (C + D) was significantly reduced, as presented in Table 2. In addition, DNA fragmentation and sperm morphology were significantly improved in Group B compared with Group A, as shown in Table 3.

**Table 1:** Patients’ characteristics.

Variable	Mean
Age	37.67 ± 5.36
Abstinent	4.00 ± 1.15
BMI	28.20 ± 3.49
Volume	2.67 ± 1.19

*Note:* BMI: body mass index (kg/m^2^).

**Table 2:** Comparison of sperm quality between preprocessing, swim‐up without secretome (Group A), and swim‐up with secretome (Group B).

Variable	Pre	Group A	Group B	*p* value
Concentration (mil/mL)	36.2 ± 18.5	36.3 ± 18.4	36.6 ± 19.3	0.995
Rapid progressive (A, %)	0.48 ± 1.32^a^	13.7 ± 8.3^b^	17 ± 8.3^c^	< 0.001
Slow progressive (B, %)	45.3 ± 11.9^a^	82.8 ± 7.1^b^	80.5 ± 7.8^b^	< 0.001
Nonprogressive + immotile (C + D, %)	54.2 ± 12.18^a^	3.4 ± 2.94^b^	2.4 ± 1.3^b^	< 0.001
Total motility (A + B, %)	39.4 ± 9.8^a^	88.5 ± 2.9^b^	97.5 ± 1.3^c^	< 0.001

*Overall sperm quality classification*				
Good (total (n; %))	—	42 (60.6%)	45 (82.7%)	—
Moderate (total (n; %))	—	3 (25.1%)	1 (17.2%)	—
Severe (total (n; %))	—	1 (14.2%)	—	—

*Note:* Data are presented as mean ± SD. A *p* value < 0.05 was considered statistically significant. Different superscript letters (a–c) indicate statistically significant differences between groups (Duncan post hoc test, *p* < 0.05).

**Table 3:** Comparison between normal and secretome addition.

Parameter	Group A (control) mean ± SD	Group B (secretome) mean ± SD	*p* value (ANOVA)
Concentration (mil/mL)	35.78 ± 18.4	35.74 ± 19.3	0.972
Total motility (A + B) (%)	88.12 ± 2.9	97.52 ± 1.3	0.003
DNA fragmentation index (%)	4.39 ± 1.1	3.48 ± 0.9	0.024
Morphology (%)	3.35 ± 0.8	4.27 ± 1.0	0.013

*Note:* Data are presented as mean ± SD. A *p* value < 0.05 was considered statistically significant.

## 4. Discussion

The significance of sperm processing can be seen by the fact that IUI and IVF are the two most critical methods of ART for achieving pregnancy and overcoming infertility. Sperm processing is engaged in the selection of viable sperm from semen samples. Direct swim‐up, conventional swim‐up, and density gradient centrifugation are among the conventional methods of sperm processing. Nonetheless, these methods only select sperm that are inherently of high quality. Consequently, we are interested in identifying methods to enhance the quality of sperm during the processing procedure. The capacity of stem cells (SCs) to self‐renew, differentiate, and produce various paracrine factors to regenerate damaged or injured cells and replenish the afflicted germ cells is a promising development in the treatment of infertile patients [4].

During the washing process, the ejaculate’s sperm and other components settle to the bottom of the tube, producing a pellet that excludes the seminal plasma. During the incubation period of the swim‐up technique, this procedure increases the contact of spermatozoa with other cells, which can result in the production of uncontrolled ROS. The sperm membrane is rich in polyunsaturated fatty acids (PUFAs). The interaction between ROS and the membrane can result in the formation of lipid electrophiles, which in turn leads to lipid peroxidation. The ability of sperm to fertilize may be reduced because of lipid peroxidation, which can impair the DNA integrity of the membrane [3]. Numerous physiological systems, including the testes, may be impaired by elevated ROS levels, which may result in a reduction in testosterone production and a disruption of spermatogenesis.

SCs can secrete extracellular vesicles (EVs) that contain proteins, lipids, RNAs (mRNA, miRNA, and lncRNA), and other biomolecules that are essential for paracrine signaling. EVs can transfer a variety of molecules and are a snapshot of the secreting cells, containing the secretome of the cell of origin. This makes them a potential treatment for a variety of reproductive disorders [4]. The secretome, which is composed of growth factors and cytokines derived from human umbilical cord mesenchymal stem cells (HUC‐MSCs), is involved in cell regeneration through a paracrine mechanism. The umbilical cord is a noninvasive source that is more accessible and has fewer ethical constraints. Additionally, it has a higher proliferative potential than bone marrow and other sources [5]. These factors have the potential to restore tissues that have been damaged by a variety of degenerative disorders. They can also serve as effective regenerative agents for the regeneration of β‐cells in the pancreas in type 1 diabetes mellitus and for the regeneration of skin in the wound healing process of burns. The regeneration of spermatogenic and Leydig cells may be facilitated by a high dose secretome. A rise in sperm motility and number was observed in the high‐dose group 1 week following secretome injections. Secretomes can migrate, proliferate, and differentiate within tissues, thereby facilitating regenerative processes. The secretome contains growth factors and cytokines that are responsible for the regeneration of spermatogenic, Sertoli, and Leydig cells in the seminiferous tubules [6].

Research has demonstrated that HUC‐MSCs have a protective effect on sperm survival, thereby contributing to the recipient testicular SC niche. The intravenous injection of HUC‐MSCs into rodents that have undergone testicular torsion and detorsion operations has the potential to inhibit ischemia/reperfusion (I/R) injury, enhance the proliferation and differentiation of spermatogonia, and facilitate the survival and regeneration of spermatogenic cells and sperm. Furthermore, the inflammatory factors and neutrophil infiltration in the testes were diminished by the treatment. Anti‐inflammatory cytokines, including TGF‐β and IL‐10, were identified in HUC‐MSCs and are recognized for their ability to dampen inflammatory responses. Cytokine secretion and T cell activation are inhibited by IL‐10, which has the capacity to regulate the inflammatory immune response. Leading to a reduction in the inflammatory response, TGF‐β induces macrophage M2–like polarization [7]. The immunosuppressive effects of HUC‐MSCs on spermatogenic cell injury may be facilitated by these anti‐inflammatory cytokines, which reduce the rate of DNA damage caused by ROS and inhibit lipid peroxidation in sperm cells [8]. HUC‐MSCs’ high‐dose secretome enhanced the motility and sperm count of rats with cisplatin‐induced testicular dysfunction as well as facilitated the regeneration of seminiferous tubules [6].

Male infertility can result from disruptions in the oxidant–antioxidant system, which can retard testicular growth and disrupt spermatogenesis, while the physiological levels of ROS assist in the regulation of normal spermatogenesis processes [9]. Melatonin, an antioxidant, may partially mitigate the oxidative damage caused by PTX, thereby safeguarding the quality of sperm [10]. The application of MSCs in reproductive therapy has become a popular area of research, in addition to antioxidants and other exogenous medications. MSCs have the capacity to renew and differentiate. They are crucial in the repair of cell damage, the prevention of cellular senescence, and the prevention of anti‐inflammatory and antioxidative damage processes [11]. Busulfan‐induced testicular damage can be substantially mitigated by MSCs derived from umbilical cords (UC‐MSCs), bone marrow (BM‐MSCs), and human amnion membranes (hA‐MSCs) [12–14]. Anti‐inflammatory and immune‐modulatory pathways, as well as modulatory effects on oxidative stress, have been demonstrated to protect rodent testes [15]. The reduction of ER stress and apoptosis by hA‐MSCs could potentially enhance the treatment of ionized radiation–induced testicular injury [16]. The background information provided by these studies is crucial for the further investigation of the application of MSCs in male reproductive health.

In addition to antioxidants and other exogenous medications, the application of MSCs in reproductive therapy has become a rapidly growing field of research. MSCs have the capacity to self‐renew and proactively differentiate. They are crucial in the repair of cell damage, immunity to cellular senescence, and the prevention of anti‐inflammatory and antioxidative damage processes [11]. In this study, we examined the efficacy of secretome in protecting males from PTX‐induced reproductive injury by analyzing the spermatogenesis processes of germ cell proliferation and meiosis, as well as the fertility potential of sperm in vivo and in vitro. In addition to promoting germ cell proliferation, secretome has the potential to restore defects in sperm fertility that have been induced by PTX, maintain sperm quality, and resist the oxidative damage caused by PTX [17]. These discoveries offer precious insights for future investigations into the role of MSCs in male reproduction.

The significant enhancement in RP motility in Group B (swim‐up with secretome) in comparison to both Group A (swim‐up without secretome) and the pretreatment samples was one of the most notable outcomes of this study. The secretome’s capacity to improve sperm functionality, which is essential for successful fertilization, is illustrated by the increase in RP motility from 0.48% (pretreatment) to 17% (Group B). Additionally, the secretome’s efficacy in optimizing sperm movement, a critical factor in the fertilization and reaching of the ovum, is underlined by the substantial increase in TM in Group B (97.52%) compared to Group A (88.12%). The bioactive factors in the secretome, such as cytokines, growth factors, and EVs, may be responsible for the observed increase in motility [18]. These components are recognized for their ability to improve the motility of sperm cells by reducing oxidative stress and promoting cellular energy metabolism [19].

DNA integrity is a critical factor in the quality of sperm, as high levels of DNA fragmentation are linked to adverse embryonic development and decreased fertilization rates [20]. The DFI in Group B was significantly lower (3.48%) than that in Group A (4.39%) in this study. This implies that the incorporation of secretome not only enhances motility but also safeguards the genetic material contained within the sperm [21]. Although the reduction in DFI may be associated with improved sperm cellular integrity, the specific antioxidant or molecular mechanisms were not evaluated in this study and required further investigation. Therefore, the biological pathways underlying these improvements remain to be clarified [22].

It is interesting that the concentration of sperm did not seem to be substantially impacted by the secretome addition in any of the groups. This implies that the secretome affects motility and DNA integrity in a positive manner, but it does not affect the number of sperm retrieved during the swim‐up process [23]. This discovery corresponds with the comprehension that the swim‐up technique primarily selects motile sperm rather than increasing the total concentration of sperm. The potential of the secretome as an adjunct in ARTs is underscored by the substantial enhancements in DNA integrity and motility that were observed in this study. The improvements observed in motility and DNA integrity suggest that secretome may serve as a supportive adjunct in ARTs, particularly in cases of suboptimal sperm quality. However, its direct influence on fertilization or pregnancy outcomes remains to be confirmed through further clinical evaluation [24, 25].

In a study on adipose‐mesenchymal stem cells (AD‐MSCs), it was demonstrated that a 24‐hour incubation of human sperm resulted in reduced sperm vacuolization and DNA fragmentation, while preserving motility and viability [26]. AD‐MSCs secrete bioactive growth factors, including VEGF, granulocyte colony–stimulating factor (G‐CSF), IGF‐1, HGF, NGF, and FGF, which stabilize membrane proteins and enhance sperm motility. Additionally, they secrete anti‐inflammatory mediators IL‐10 and TGF‐β and protective enzymes like SOD [2]. Studies conducted in the past on the Cumulus Cell‐secretome (CCs) have shown that they have a higher motility (65%), normal morphology (around 9%), and concentration (approximately 75 × 10^6^/mL) than the swim‐up (normal) group (without CCs), with a DFI range of 11%. CCs also exhibited a substantial increase in oxygen consumption, mitochondrial membrane potential, ATP content, the proportion of motile sperm, and sperm velocity, which may have alleviated the effects of ROS [27, 28].

To the best of our knowledge, this is the first study to evaluate the direct in vitro effects of secretome on human ejaculated sperm. The significant improvements observed in motility, morphology, and DNA integrity indicate that secretome may serve as a supportive adjunct in sperm preparation for ARTs. In contrast, other studies have investigated the effects of intravenously administered HUC‐MSCs on dysfunctional testes in rodents [29, 30]. Compared to discontinuous density gradient centrifugation, sperm washing followed by swim‐up has been shown to yield higher morphology recovery rates and lower sperm concentration recovery rates [3]. The discrepancies may be due to factors such as semen quality and differences in sperm preparation techniques [31, 32]. Furthermore, this investigation encompassed samples with aberrant sperm concentrations (oligozoospermia) and motility (asthenozoospermia), unlike other studies that analyzed normozoospermic samples [1]. Additional research is required to determine whether the observed in vitro improvements in sperm motility, morphology, and DNA integrity translate into measurable clinical outcomes, such as fertilization, embryo development, and pregnancy rates. The present findings should therefore be interpreted as preclinical evidence supporting the potential use of secretome as an adjunct during sperm preparation for assisted reproduction. A prospective clinical study is currently being designed to evaluate fertilization rate, embryo quality, and pregnancy outcomes in IVF cycles using secretome‐treated sperm. Furthermore, larger studies including more diverse patient populations are required to confirm reproducibility and optimize conditions for clinical implementation. Future studies should include mechanistic analyses, such as measurements of ROS, mitochondrial membrane potential, and acrosome reaction, to further elucidate the biological mechanisms underlying the improvements observed in sperm motility and DNA integrity.

## 5. Conclusions

Secretome demonstrated beneficial effects by increasing the proportion of RP motility and TM, decreasing the percentage of sluggish and immotile sperm, reducing DNA fragmentation rates, and enhancing sperm morphology. The findings indicate its potential value as a supportive component in sperm preparation methods; however, further mechanistic and clinical studies are required to confirm whether these improvements translate into enhanced fertilization or pregnancy outcomes.

## Author Contributions

Binarwan Halim, Laura Agnes Edessa Sibuea, Carine Annabella Widjaja, and Diana Novia had full access to all study data and took responsibility for its integrity and accuracy. Concept and design, data acquisition, analysis, interpretation, drafting, and supervision were conducted by Binarwan Halim, Laura Agnes Edessa Sibuea, Carine Annabella Widjaja, and Diana Novia. Dr. Cynthia Retna Sartika provides and supports the secretome product used in this study. Mrs. Rima Haifa and Mrs. Karina Kalasuba contributed to the submission and revision of the manuscript. Binarwan Halim, Laura Agnes Edessa Sibuea, Carine Annabella Widjaja, and Diana Novia contributed equally and should be considered co‐first authors.

## Funding

This work was funded by PT Prodia StemCell Indonesia.

## Ethics Statement

The authors have nothing to report.

## Consent

The authors have nothing to report.

## Conflicts of Interest

The authors declare no conflicts of interest.

## Data Availability

The data used to support the findings of this study are included within the article. The raw data that support the findings of this study are available on request from the corresponding author, upon reasonable request.

## References

[1] Definisi Infertilitas Dan Keguguran Berulang: Pendapat Komite, Fertility and Sterility. (2020) 113, no. 3, 533–535, 10.1016/j.fertnstert.2019.11.025. 32115183

[2] Angellee J. , Novalinda Ginting C. , Chiuman L. , and Halim dan B. , Peran Plasma Kaya Trombosit Terhadap Kualitas Sperma Pada Pasangan Pria Yang Menjalani Perawatan Infertilitas, *Dalam InHeNce 2021-2021 IEEE International Conference on Health, Instrumentation and Measurement, and Natural Sciences*, 2021, Institute of Electrical and Electronics Engineers Inc, 10.1109/InHeNce52833.2021.9537240.

[3] Bader R. , Ibrahim J. N. , Moussa M. et al., Efek in Vitro Plasma Kaya Trombosit Autolog Pada Stres Oksidatif Yang Diinduksi H2O2 Pada Spermatozoa Manusia, Andrologia. (2020) 8, no. 1, 191–200, 10.1111/andr.12648, 2-s2.0-85065715081.

[4] Qamar A. Y. et al., P, eran Sel Punca Dan Vesikel Ekstraseluler Yang Berasal Darinya Dalam Memulihkan Kesuburan Wanita Dan Pria. (2021) MDPI.

[5] Yan L. , Zhou L. , Yan B. et al., Efek Menguntungkan Berbasis Faktor Pertumbuhan Dari Lisat Trombosit Pada Sel Punca Yang Berasal Dari Tali Pusat Dan Penggunaan Sinergisnya Dalam Pengobatan Osteoartritis, Cell Death & Disease. (2020) 11, no. 10, 10.1038/s41419-020-03045-0.

[6] Prihatno S. A. , Padeta I. , Larasati A. D. et al., Pengaruh Sekretom Pada Disfungsi Testis Yang Diinduksi Cisplatin Pada Tikus, Veterinary World. (2018) 11, no. 9, 1349–1356, 10.14202/vetworld.2018.1349-1356, 2-s2.0-85055351587. 30410245 PMC6200560

[7] Arfianti A. , Ulfah U. , Hutabarat L. S. et al., Hipoxia Memodulasi Sekresi Faktor Pertumbuhan Sel Punca Mesenkimal Yang Berasal Dari Tali Pusat Manusia, Biomedicine. (2023) 13, no. 3, 49–56, 10.37796/2211-8039.1416. 37937056 PMC10627211

[8] Zhong L. , Yang M. , Zou X. , Du T. , Xu H. , and Sun dan J. , Sel Stroma Mesenkimal Multipotensi Tali Pusat Manusia Meringankan Cedera iskemia-reperfusi Akut Sel Spermatogenik Melalui Pengurangan Respons Inflamasi Dan Stres Oksidatif, Stem Cell Research & Therapy. (2020) 11, no. 1, 10.1186/s13287-020-01813-5.

[9] Bader R. , Ibrahim J. N. , Mourad A. et al., Peningkatan Vakuolisasi Sperma Manusia Dan Fragmentasi DNA Yang Dikultur Bersama Dengan Sekretom Sel Punca Mesenkimal Yang Berasal Dari Lemak: Efek in Vitro, Jurnal Internasional Sel Punca. (2019) 12, no. 3, 388–399, 10.15283/ijsc19047.

[10] Hosseini S. , Kazemi M. , Salehpour S. , Saharkhiz N. , and Majdi L. , Evaluation of the Effect of Platelet-Rich Plasma (PRP) on the Sperm Parameters, Taiwanese Journal of Obstetrics & Gynecology. (2025) 64, no. 2, 313–318, 10.1016/j.tjog.2024.12.010. 40049817

[11] Han X. , Liao R. , Li X. et al., Mesenchymal Stem Cells in Treating Human Diseases: Molecular Mechanisms and Clinical Studies, Signal Transduction and Targeted Therapy. (2025) 10, no. 1, 10.1038/s41392-025-02313-9. PMC12371117 40841367

[12] Organisasi Kesehatan Dunia, 2024.

[13] Guo X. , Liu B. , Xu P. et al., Research on the Mechanism of Human Umbilical Cord Mesenchymal Stem Cells and Their Extracellular Vesicles in the Treatment of Common Reproductive Diseases, Stem Cell Research & Therapy. (2025) 16, no. 654, 10.1186/s13287-025-04773-w. PMC12639902 41272688

[14] Zhong L. , Yang M. , Zou X. , Du T. , Xu H. , and Sun J. , Human Umbilical Cord Multipotent Mesenchymal Stromal Cells Alleviate Acute ischemia-reperfusion Injury of Spermatogenic Cells via Reducing Inflammatory Response and Oxidative Stress, Stem Cell Research & Therapy. (2020) 11, no. 1, 10.1186/s13287-020-01813-5. PMC7366899 32680554

[15] Peng L. , Chen X. , Gao F. et al., Human Umbilical Cord Mesenchymal Stem Cell-Derived Exosomes Rescue Testicular Aging, Biomedicines. (2024) 98, no. 1, 10.3390/biomedicines12010098. PMC10813320 38255205

[16] Cetinkaya-Un B. , Un B. , Akpolat M. , Andic F. , and Yazir Y. , Human Amnion Membrane-Derived Mesenchymal Stem Cells and Conditioned Medium Can Ameliorate X-Irradiation-Induced Testicular Injury by Reducing Endoplasmic Reticulum Stress and Apoptosis, Reproductive Sciences. (2022) 29, no. 3, 944–954, 10.1007/s43032-021-00753-6. 34642916

[17] Yan L. , Zhou L. , Yan B. et al., Growth Factors-based Beneficial Effects of Platelet Lysate on Umbilical cord-derived Stem Cells and Their Synergistic Use in Osteoarthritis Treatment, Cell Death & Disease. (2020) 11, no. 10, 10.1038/s41419-020-03045-0. PMC7560841 33057008

[18] Qamar A. Y. , Hussain T. , Rafique M. K. et al., The Role of Stem Cells and their Derived Extracellular Vesicles in Restoring Female and Male Fertility, Cells. (2021) 10, no. 9, 10.3390/cells10092460. PMC8468931 34572109

[19] Practice Committee of the American Society for Reproductive Medicine , Definitions of Infertility and Recurrent Pregnancy Loss: a Committee Opinion, Fertility and Sterility. (2013) 99, no. 1, 10.1016/j.fertnstert.2012.09.023, 2-s2.0-84871923467. 23095139

[20] Zhang Y. , Liu Y. , Teng Z. et al., Human Umbilical Cord Mesenchymal Stem Cells (hUC-MSCs) Alleviate paclitaxel-induced Spermatogenesis Defects and Maintain Male Fertility, Biological Research. (2023) 56, no. 1, 10.1186/s40659-023-00459-w. PMC10424423 37574561

[21] Jordana Mashiach M. D. , Khaled Zohni M. D. , Lianet Lopez M. Sc et al., Human Umbilical Cord Perivascular Cells Prevent Chemotherapeutic drug-induced Male Infertility in a Mouse Model, F&S Science. (2021) 2, no. 1, 24–32, 10.1016/j.xfss.2020.12.002. 35559762

[22] Yaghoubi Y. , Movassaghpour A. A. , Zamani M. , Talebi M. , Mehdizadeh A. , and Yousefi M. , Human Umbilical Cord Mesenchymal Stem Cells derived-exosomes in Diseases Treatment, Life Sciences. (2019) 233, 10.1016/j.lfs.2019.116733, 2-s2.0-85070202436. 31394127

[23] Andrea O. M. D. , Andreas Obruca M. D. , Michaela P. M. D. et al., Vascular Endothelial Growth Factor and Its Receptors in Male Fertility, Fertility and Sterility. (1999) 72, no. 2, 269–275, 10.1016/s0015-0282(99)00234-4, 2-s2.0-0033180063. 10438994

[24] Umer A. , Khan N. , Geene D. L. , Habiba U. E. , Shamim S. , and Khayam A. U. , The Therapeutic Potential of Human Umbilical Cord Derived Mesenchymal Stem Cells for the Treatment of Premature Ovarian Failure, Stem Cell Reviews and Reports. (2023) 19, no. 3, 651–666, 10.1007/s12015-022-10493-y. 36520408 PMC10070285

[25] Yang R. F. , Liu T. H. , Zhao K. , and Xiong C. L. , Enhancement of Mouse germcell-associated Genes Expression by Injection of Human Umbilical Cord Mesenchymal Stem Cells into the Testis of chemical-induced Azoospermic Mice, Asian Journal of Andrology. (2014) 16, no. 5, 698–704, 10.4103/1008-682x.129209, 2-s2.0-84907013693. 24830694 PMC4215652

[26] Gauthier-Fisher A. , Kauffman A. , and Librach C. L. , Potential Use of Stem Cells for Fertility Preservation, Andrology. (2019) 8, no. 4, 862–878, 10.1111/andr.12713. 31560823

[27] Shlush E. , Maghen L. , Swanson S. et al., In Vitro Generation of Sertoli-like and Haploid Spermatid-like Cells from Human Umbilical Cord Perivascular Cells, Stem Cell Research & Therapy. (2017) 8, no. 1, 10.1186/s13287-017-0491-8, 2-s2.0-85013243997. PMC5312448 28202061

[28] Arigue A. D. , Zienab A. G. , Mona A. A. et al., Hypoxia-Preconditioned Human Umbilical Cord Blood-Derived Mesenchymal Stem Cells Mitigate Hypoglycemic Testicular Injury Induced by Insulin in Rats, Cells Tissues Organs. (2020) 209, no. 3, 83–100, 10.1159/000510363. 33113534

[29] Silva G. H. , Torres A. S. L. , Rosas M. R. et al., Effects of Semen Processing on Sperm Function: Differences Between Swim-Up and Density Gradient Centrifugation, World J Mens Health. (2020) 19, no. 4, 740–749, 10.5534/wjmh.200115. PMC8443982 33474848

[30] Kari L. R. , Harold F. R. , Gloria C. , Hakkarainen R. M. , Anchan G. L. , and Mutte W. A. , Sperm Processing for Advanced Reproductive Technologies: where are We Today?, Biotechnology Advances. (2016) 34, no. 5, 578–587, 10.1016/j.biotechadv.2016.01.007, 2-s2.0-84958206638. 26845061

[31] Erica T. Y. L. , Cheuk L. L. , Xinyi T. et al., Simulating Nature in Sperm Selection for Assisted Reproduction, Nature Reviews Urology. (2022) 19, 16–36, 10.1038/s41585-021-00530-9. 34741158

[32] Nazl C. , Cihan K. , Umit C. , Tahir T. , and Gulcin A. M. , Retrospective Comparison of the Semen Preparation Techniques for Intrauterine Insemination: Swim-Up Versus Density Gradient Method, Journal of Gynecology Obstetrics and Human Reproduction. (2022) 51, no. 3, 10.1016/j.jogoh.2022.102321. 35063717

